# Sickle cell disease knowledge, attitudes, and management practices among nurses in northern Ghana: a cross-sectional study

**DOI:** 10.3389/fpubh.2026.1811313

**Published:** 2026-07-13

**Authors:** Mubaric Yakubu, Yula Salifu, Sarah Damten Kombian, Abdul-Sabur Mohammed Suglo, Torjim Salifu, Joseph Lasong, Ruth Nimota Nukpezah

**Affiliations:** 1 Department of Nutritional Sciences, School of Allied Health Sciences, University for Development Studies, Tamale, Ghana; 2 Department of Biochemistry and Biotechnology, Kwame Nkrumah University of Science and Technology, Kumasi, Ghana; 3 Department of Population and Reproductive Health, School of Public Health, University for Development Studies, Tamale, Ghana; 4 Pediatric Nursing Specialist Program, Ghana College of Nurses’ and Midwives’, Accra, Ghana; 5 Department of Social and Behavioral Change, School of Public Health, University for Development Studies, Tamale, Ghana; 6 Department of Preventive Health Nursing, School of Nursing and Midwifery, University for Development Studies, Tamale, Ghana

**Keywords:** attitudes, Ghana, knowledge, nurses, pediatric, practice, sickle cell disease

## Abstract

**Background:**

Sickle cell disease (SCD) is a global public health concern with high morbidity and mortality among children, particularly in low-and-middle-income countries. Affected children require specialized care, and nurses bear major responsibility for their management. This study assessed nurses’ knowledge of SCD and their attitudes and practices towards its management in the pediatric unit of Tamale Teaching Hospital.

**Methods:**

This hospital-based cross-sectional study randomly sampled 147 nurses caring for SCD clients from February to April 2024. Data was analyzed using SPSS and STATA. Multiple linear regression identified factors associated with SCD management practices (*p* ≤ 0.05).

**Results:**

In all, most respondents (55.8%) demonstrated low SCD knowledge, despite 90.5% knew SCD is caused by genetic mutation. Mean attitude score was 24.40 ± 5.30 (range 12–40); 36.1% agreed that clients with SCD are sometimes perceived as drug-seeking. Age (*β* = 0.32, *p* = 0.013), knowledge level (*β* = 1.75, *p* = 0.001), and attitudes (*β* = −0.21, *p* = 0.006) were associated with practice.

**Conclusion:**

Nurses demonstrated low SCD knowledge, neutral attitudes, and good practices. Age and knowledge positively associated with practice, while attitudes showed negative association. These deficiencies may adversely affect client outcomes. Training workshops and behavior change communication across the nursing fraternity could improve SCD management practices.

## Introduction

Sickle-cell disease (SCD) is a worldwide health problem, affecting many countries and ethnic groups, and was declared a public health priority in 2006 (1). SCD is an inherited disorder of the hemoglobin (Hb) molecule of the red blood cells (RBCs) that is associated with serious complications reduced life expectancy and low quality of life (2, 3). Globally, there are over 300,000 births/year of children with SCD and over 75% of people with SCD living in Sub-Saharan Africa (SSA), which is projected to increase to 85% by the year 2050 (3). Nurses play a crucial role in managing and improving the quality of life of individuals with SCD (4). Stewart et al. (5) suggests that nurses in pediatric or emergency units may have more negative attitudes about managing pain among clients with SCD than hematologists. According to a previous study, while nurses were educated about the management of crises and problems linked with SCD, they were less aware of client health education (6). Furthermore, a study carried out in the Jazan region found that healthcare staff, notably nurses, had negative attitudes toward clients with SCD (7). People with SCD may suffer because of these attitudes, which can lead to poorer care and outcomes. A study conducted in Dar es Salaam, Tanzania highlighted that there was a general lack of knowledge on SCD among nurses (3). Clients with SCD face unique healthcare challenges that require specialized attention and care. Nurses play a pivotal role in the management of such clients. Oti et al. (2) indicate that nurses must assume the role of advocates, educators, and case managers after clients have been diagnosed with SCD. The nurse has a major responsibility of knowing about the disease itself, how to approach each client; and using appropriate management and interventions for SCD. In recent years, more research has been reported on the epidemiologic features of acute pain episodes, as well as on the psychological and social characteristics of clients with SCD who experience frequent pain episodes (3). Little is known about healthcare professionals’ attitudes and practices towards clients with SCD. Thus, this study sought to assess the knowledge of nurses on sickle cell disease and their attitudes and practices towards its management in the pediatric unit of the Tamale Teaching Hospital.

## Materials and methods

### Study area

The study was conducted in the pediatric unit of the Tamale Teaching Hospital (TTH). TTH is the only teaching hospital in the northern part of Ghana and the main referral center for the country’s five northern regions. The hospital has a bed capacity of 860 and runs services such as OPD- (general out-patient and specialist out-patient services) and in-patient services. The pediatric ward of the facility attends to children up to 14 years old. It has a bed capacity of 86. The ward has a staff strength of 200 nurses, including 2 pediatric nurse specialists, 1 neonatal nurse specialist, 10 pediatric nurses, and medical doctors, including 2 pediatricians, 2 medical officers, 14 house officers, and 6 nutrition officers with 4 housekeepers.

### Study design and population

A hospital-based cross-sectional design was used to sample 147 nurses who care for client with SCD in the Tamale Teaching Hospital from 1st February to 30th April, 2024. Registered nurses who were present during the study, agreed to participate in the study and had at least 12 months of working experience and provided care to clients seeking medical services for SCD in the pediatric unit of Tamale Teaching Hospital were sampled. Rotation nurses, student nurses and registered nurses at the pediatric unit who disagreed to participate in the study were excluded.

### Sample size and sampling technique

The sample size for the study was estimated using the Yamane Taro formula ( $n=\frac{N}{1+N(e)2}$ ). Where n = desired sample size for the current study, *N* = total expected number of nurses in the pediatric ward caring for children with SCD (200), and *e* = 5% marginal error (5%). A sample size 133 was estimated for the study. After a 10% non-response rate was calculated, a final sample size of 147 was considered in the final analysis.

The study employed a simple random sampling technique to recruit respondents for the study. A complete sampling frame consisting of all nurses working in the pediatric ward was obtained in Microsoft Excel format from the ward administration. Each nurse on the list was assigned a unique identification number, after which the randomization function in Microsoft Excel was used to randomly select participants from the sampling frame.

An initial set of 50 nurses was randomly selected from the list. The randomization process was repeated in subsequent rounds to ensure that every nurse had an equal and independent chance of being selected into the study. Nurses who were selected but declined participation were replaced through the same random selection procedure from the remaining eligible nurses. This process continued until the required sample size of 147 respondents was achieved.

### Instruments and data collection process

Comprehensive literature reviews (2, 3, 8) and expert responses were used to design the face-to-face structured questionnaires (Additional file 1). The questionnaires contained socio-demographic, knowledge on SCD, attitudes towards SCD management, and nurses’ practices towards clients with SCD characteristics. The survey questions encompassed aspects such as diagnosis, treatment, and management. For each measure, scores on items showed sufficient internal consistency (reliability coefficients [Cronbach’s alpha (*α*)] of ≥0.6 for three or more items). The reliability coefficient for the knowledge, attitude and practice questions was 0.76, 0.78 and 0.81, respectively. The scale reliability coefficient for the entire data collection tool was (*α*) ≥ 0.75. Four research assistants with Master of Public Health Degrees and a background in Nursing who were fluent in English, Dagbani, and other local dialects from the University for Development Studies were recruited and trained for 3 days for the data collection process. Respondents were informed on the nature of the study and informed consent was sought prior to the data collection. Those who consented and could self-administer the survey questions were given the survey forms to complete. The survey was conducted in English Language, since the population was highly literate. In cases where the respondents could not understand some questions, research assistants clarified each response given to each survey question to the satisfaction of the respondents. The data collection process was supervised by the first and second author. The study followed the STROBE guidelines in reporting data for cross-sectional surveys (9).

Questionnaires were pretested among 15 nurses who care for clients with SCD at the pediatric unit of the Tamale SDA Hospital, a hospital within the Tamale Metropolis which bears similar characteristics to the study setting. The pretested data was not included in the final analysis. The survey questions were adjusted for comprehensiveness (involving reordering of some questions) on a day-to-day basis after pretesting the questions, to improve consistency and coherence of the tools. Interviews lasted about 30 min.

### Measures

To assess knowledge on SCD, 10 multiple-choice questions were used, where each correct answer was scored one mark and incorrect answers received zero marks. This was obtained from a related literature (6). The total score for SCD knowledge was calculated by summing the marks for each item, and the mean ± standard deviation (SD) of the SCD knowledge score was computed. Knowledge level was categorized into low and high. Respondents whose knowledge level score was above the mean score were grouped as having high knowledge levels while those with ≤ the mean score were categorized as having low knowledge levels. Attitudes of nurses towards the diagnosis, crisis management, and out-patient follow-up of clients with SCD were evaluated using 8 items rated on a 5-point Likert scale (1 = strongly disagree to 5 = strongly agree). The total score for attitudes towards SCD management was calculated by summing the marks for each item, and the mean ± standard deviation (SD) of the SCD management attitudes score was computed. Nurses’ practices related to the diagnosis, crisis management, and out-patient follow-up of clients with SCD were assessed using a 28-item questionnaire where responses were recorded as ‘yes’ or ‘no’. Each correct response was awarded one mark, and incorrect responses received zero marks. The mean ± SD score of nurses’ practices in these areas was computed based on their responses.

### Data analysis

All data were entered and cleaned in Microsoft Excel and imported into SPSS (version 25.0) and STATA/IC 14 for analysis. Descriptive statistics were produced for frequencies, percentages, mean scores and standard deviations. Data were normally distributed upon examination with a histogram for the dependent variable. To test for differences in means between categorical and continuous variables, independent Student’s t-test and one-way ANOVA were employed. Correlation analysis was used to examine linear relationships among continuous variables. Additionally, a multiple linear regression model was developed to explore the association between the independent factors and the dependent variable (nurses’ practices related to the diagnosis, crisis management, and out-patient follow-up of Clients with SCD). Statistical significance was set at a *p*-value of less than 0.05 with a 95% confidence interval.

## Results

### Socio-demographic characteristics of the respondents

The Table 1 below highlights the socio-demographic characteristics of the respondents. More than half (55.8%) of the nurses were females. The mean age of the respondents was 31.9 ± 5.2 years. Majority (55.1%) of the respondents had a bachelor’s degree, with a mean year of working of 6. 3 ± 4.7 years (Table 1).

**Table 1:** Socio-demographic and general characteristics of the respondents.

Variable	Frequency	Percentage
Age (mean age of 31.9 SD 5.2)		
20–30	70	47.6
31–40	69	46.9
41–50	8	5.4
Gender		
Male	65	44.2
Female	82	55.8
Level of education		
Diploma degree	61	41.5
Bachelor’s degree	81	55.1
Master’s degree	5	3.4
Marital status		
Married	88	59.9
Singled	59	40.1
Years of working (mean working yrs 6.3 SD 4.7)		
<5 years	85	57.8
5–10 years	32	21.8
>10 years	30	20.4

SD, standard deviation.

### Level of knowledge among nurses about sickle-cell disease and its management

Out of the ten (10) questions, the respondents scored less than 50% in four (4) questions. Despite the high knowledge (90.5%) on the cause of sickle disease among the respondents and the most common symptoms of SCD (59.9%). Only 18.4% knew that silent cerebral infarcts can put clients with SCD at increased risk for stroke, 29.3% knew how much fluids should clients with sickle cell disease drink and 27.9% knew hydroxyurea is a medication used to treat sickle cell disease. The Mean ± SD score of knowledge level was 5.27 ± 1.65 with a minimum score of 0.0 and a maximum score of 9 (Table 2).

**Table 2:** Knowledge level among nurses on sickle-cell disease and its management.

Knowledge variable	Correct response	
	Frequency	Percentage
Sickle disease is caused by	133	90.5
The most common symptoms of sickle cell disease include	88	59.9
How often should people with sickle cell disease have routine health screening	119	81.0
Which medication is most commonly used to manage sickle cell	96	65.3
What put clients with sickle cell disease at increased risk for stroke	27	18.4
Chronic kidney disease is a common complication of sickle cell	80	54.4
What percentage of clients with sickle disease have gallstones by age 20	5	3.4
How much fluids should clients with sickle cell disease drink	30	20.4
Hydroxyurea is a medication used to treat sickle cell disease	41	27.9
All of the following can trigger a sickle cell pain crisis except	93	63.3

### Overall knowledge level of nurses about sickle-cell disease and its management

**Figure 1:**
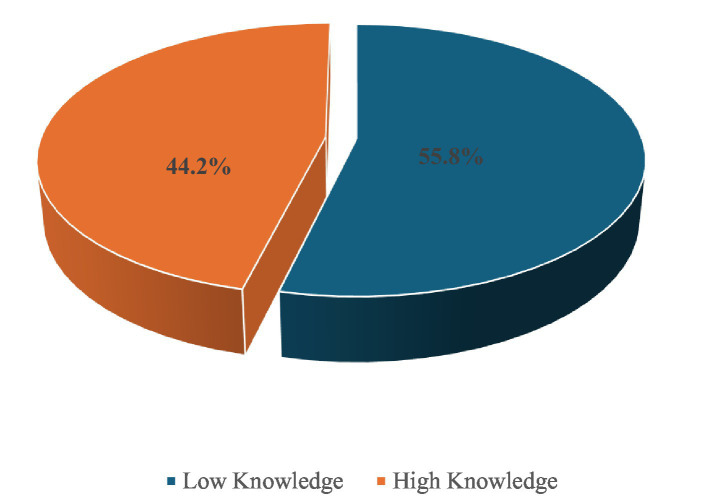
Overall knowledge level of nurses on sickle-cell disease and its management.

High knowledge was categorized as respondents who scored 75% and above and low knowledge was categorized as lower than 75% of the total knowledge score. In all majority (55.8%) of the respondents demonstrated low knowledge on SCD and its management whereas 44.2% demonstrated high knowledge on SCD and its management (Figure 1).

### Attitudes of nurses towards clients with sickle-cell disease and its management

About 36.1% of the respondents agreed that clients with sickle cell disease are sometimes perceive as drug sicken. Furthermore, 12.9% of the respondents agreed that sickle cell disease is often associated with low socioeconomic status. Similarly, 34.0% of the respondents agreed that SCD receives less research funding and attention. The mean ± SD attitude of the nurses towards clients with sickle-cell disease and its management was 24.40 ± 5.30 with a minimum score of 12 point and maximum score of 40 points (Table 3). This reflects their neutral attitudes towards the practice and management of SCD.

**Table 3:** Attitudes of nurses towards clients with sickle-cell disease and its management.

Variable	SD	D	N	A	SA	Mean (SD)
Clients with sickle cell disease are sometimes perceive as drug sicken	13 (8.8)	26 (17.7)	20 (13.6)	53 (36.1)	33 (22.4)	3.46 (1.26)
Sickle cell disease is often associated with low socioeconomic status	50 (34.0)	38 (25.9)	25 (17.0)	19 (12.9)	14 (9.5)	2.39 (1.33)
Clients with sickle cell disease have a tendency to be non-compliant	13 (8.8)	36 (24.5)	18 (12.2)	56 (38.1)	23 (15.6)	3.28 (1.24)
Sickle disease primarily affect Africans	6 (4.1)	24 (16.)	34 (23.1)	52 (35.4)	30 (20.4)	3.50 (1.12)
There are negative stereotypes association with sickle disease	8 (5.4)	20 (13.6)	21 (14.3)	60 (40.8)	33 (22.4)	3.61 (1.15)
Sickle cell disease receives less research funding and attention	9 (6.1)	26 (17.7)	40 (27.2)	50 (34.0)	21 (14.3)	3.33 (1.11)
Discrimination against clients with sickle cell disease still exist in the health care system	29 (19.7)	32 (21.8)	16 (10.9)	51 (34.7)	16 (10.9)	2.94 (1.36)
I make a conscious effort to avoid stereotyping clients with sickle cell disease	10 (6.8)	11 (7.5)	18 (12.2)	46 (31.3)	61 (41.5)	3.93 (1.27)

SD_***x***_, strongly disagree, D, disagree, A, agree, SA, strongly agree, N, neutral, SD, standard deviation.

### Practices of nurses when caring for clients with sickle-cell disease

Majority (84.8%) of the respondents do haemoglobin profile. Almost all the respondents are able to diagnose pain (93.2%), fever (75.5%), swelling of hands and feet in infants (68.7%), jaundice (70.7%) and notion of multiple deaths in the immediate family (48.3%). About 90.5% of nurses assessed pain based on client complaint whiles 83.3% uses a scale to assessed pain. Further, 93.2% of nurses used transfusion to managed clients with SCD, 87.8% used hydration with saline, 82.3% used oxygen and 84.4% used hydroxyurea. The mean ± SD practice score was 21.27 ± 5.91 with a minimum score of 3 point and maximum score of 28 points (Table 4).

**Table 4:** Practices of nurses when caring for clients with sickle cell disease.

Statement	Yes (%)	No (%)
Screening or diagnosis		
Haemoglobin profile	124 (84.4)	23 (15.6)
Practitioner diagnosed at least one case of SCD	121 (82.3)	26 (17.7)
Symptoms or signs evoking diagnosis		
Fever	111 (75.5)	36 (24.5)
Pain	137 (93.2)	10 (6.8)
Cutaneous-mucosal pallor	117 (79.6)	30 (20.4)
Notion of previous transfusion	103 (70.1)	44 (29.9)
Jaundice	104 (70.7)	43 (29.3)
Swelling of hands and feet in infants	101 (68.7)	46 (31.3)
Notion of multiple deaths in the immediate family	71 (48.3)	76 (51.7)
Assessment of pain		
Subjective (based on client complaint)	133 (90.5)	14 (9.5)
Using a scale	123 (83.7)	24 (16.3)
Use of usual analgesics		
Empirically	111 (75.5)	36 (24.5)
According to WHO guidelines	124 (84.4)	23 (15.6)
Analgesics used		
Paracetamol	105 (71.4)	42 (28.6)
Paracetamol and codeine	74 (50.3)	73 (49.7)
NSAIDs (diclofenac, ibuprofen…)	110 (74.8)	37 (25.2)
Tramadol	66 (44.9)	81 (55.1)
Morphine	118 (80.3)	29 (19.7)
Other treatments		
Transfusion	137 (93.2)	10 (6.8)
Hydration with saline	129 (87.8)	18 (12.2)
Oxygen	121 (82.3)	26 (17.7)
Hydroxyurea	124 (84.4)	23 (15.6)
Out-patient follow-up		
Provider has ever followed a SCD client	108 (73.5)	39 (26.5)
SCD follow-up may be once a month	116 (78.9)	31 (21.1)
Laboratory tests		
Haemoglobin. White blood cell count. Complete blood count	127 (86.4)	20 (13.6)
Lactate dehydrogenase	91 (61.9)	56 (38.1)
Urea and creatinine	120 (81.6)	27 (18.4)
Abdominal ultrasound	101 (68.7)	46 (31.3)

SCD, sickle cell disease, NSAIDS, non-steroidal anti-inflammatory drugs, WHO, World Health Organization.

### Univariate analysis of factors associated with practice score among nurses when caring for clients with SCD

Respondents who were between the ages of 41–50 years had a higher mean score of 34.75 (±3.10) years compared to those who are between the ages of 20–30 years, 19.42 (±6.7) years. Also, respondents who have master’s degrees had higher mean scores of 26.4 (±1.7) compared to those with diploma certificates, 19.26 (±6.6). The study further showed a significant association between marital status (*p* < 0.001), years of working (*p* < 0.001) and practice score when caring for clients with SCD (Table 5).

**Table 5:** Mean (SD) nurses practice score stratified by general and socio-demographic characteristics.

Variable	Frequency	Mean (SD)	*p*-value
Age			
20–30	70	19.42 (6.7)	0.001
31–40	69	22.73 (4.6)	
41–50	8	24.75 (3.1)	
Gender			
Male	65	21.16 (5.7)	0.852
Female	82	21.35 (6.1)	
Level of education			
Diploma degree	61	19.26 (6.6)	0.001
Bachelor’s degree	81	22.46 (4.9)	
Master’s degree	5	26.4 (1.7)	
Marital status			
Married	88	22.77 (4.6)	0.000
Singled	59	19.03 (6.85)	
Years of working			
<5 years	85	19.88 (6.2)	0.001
5–10 years	32	21.90 (5.3)	
>10 years	30	24.53 (3.8)	

SD, standard deviation.

### Correlation analysis among continuous variables and practice score of nurses when caring for clients with SCD

A strong positive correlation (*r* = 0.348, *p* = 0.001) was observed between nurses practice when caring for clients with SCD and number of years of working. Also, a strong positive correlation (*r* = 0.442, *p* = 0.001) was observed between nurses practice towards the management of clients with SCD and age of the respondents. However, a negative correlation (*r* = −0.232, *p* = 0.001) was observed between the nurses’ attitudes and their practice when caring for clients with SCD. Furthermore, a strong positive correlation (*r* = 0.488, *p* = 0.001) was observed between nurses practice and their knowledge on SCD (Table 6).

**Table 6:** Correlation analysis among continuous variables and practice score of nurses when caring for clients with sickle-cell disease.

Variable	Age	Number of years working	Practice score	Attitude score	Knowledge score
Age	1.000				
Number of years working	0.755^**^	1.000			
Practice score	0.442^**^	0.348^**^	1.000		
Attitude score	−0.120	−0.072	−0.232^**^	1.000	
Knowledge score	0.290^*^	0.271^**^	0.488^**^	−0.020	1.000

**Correlation is significant at *p* < 0.01 level (2-tailed), *Correlation is significant at *p* ≤ 0.05 level (2-tailed).

### Multiple linear regression analysis of factors associated with the practice of nurses when caring for clients with SCD

The independent variables accounted for 39.1% of the variability in the dependent variable. Age of the respondents showed a positive relationship with nurses’ practices when caring for clients with sickle-cell disease, indicating that a one-unit increase in age is associated with a 0.32 increase in improved nurses’ practice towards SCD management [B = 0.32, 95%CI (0.12, 0.88), *p* = 0.013]. Additionally, knowledge on SCD among respondents was positively associated with their practice towards SCD management [B = 1.75, 95%CI (1.70, 5.33), *p* < 0·001]. Specifically, a one-unit increase in SCD knowledge was associated with a 1.75 increase in better nurses’ practice towards SCD management. Conversely, nurses’ attitudes towards clients with SCD and its management demonstrated a negative relationship with their practice [B = −0.21, 95%CI (−0.44, −0.23), *p* = 0.006]. A one-unit increase in nurses’ attitudes led to a 0.21 decrease in their practice when caring for clients with SCD (Table 7).

**Table 7:** Multiple linear regression analysis of factors associated with practice of nurses when caring for clients with sickle-cell disease.

Variable	B	95% CI	Beta	T	*p*-value
Constant	9.82			2.16	0.032
Age	0.32	(0.12, 0.88)	0.28	2.52	0.013
Years of working	−0.09	(−0.10, 2.33)	−0.08	−0.72	0.475
Level of education					
Bachelor’s degree	Reference				
Diploma degree	−1.03	(−1.21, 0.72)	−0.08	−1.07	0.288
Master’s degree	3.27	(0.54, 4.33)	0.10	1.47	0.144
Marital status					
Married	Reference				
Singled	−0.71	(−0.82, 0.88)	−0.05	−0.75	0.459
Nurses’ SCD knowledge score	1.75	(1.70, 5.33)	0.39	5.66	0.000
Nurses attitude score	−0.21	(−0.44, −0.23)	−0.19	−2.77	0.006

*F* (7, 139) = 12.76, *p* < 0.0001, *R*^2^ = 0.391.

## Discussion

The present study examines nurses’ knowledge on sickle cell disease and their attitude and practice towards managing clients with sickle cell disease in the pediatric unit of the Tamale Teaching Hospital. The study reported a mean knowledge score of 5.27 out of 8, where the overall knowledge level was considered low (44.2%). This findings is consistent with a study conducted by Stewart et al. (5), who discovered that although nurses have a general understanding of SCD, there are still a lot of unanswered questions, especially when it comes to the specialized care components. According to the findings of the study, majority of the respondents (55.8%), demonstrated low knowledge of the sickle-cell disease and its management. This situation is not limited to Tamale Teaching Hospital; despite the low knowledge in the present study, it is however higher in a study conducted in Tanzania which showed that only 25.1% had good knowledge of SCD (3). However, 90.5% of nurses were aware that a genetic mutation causes sickle cell disease (SCD). This high degree of knowledge is in line with research by Phan (10) at the Johns Hopkins University, which found that medical professionals typically possess a solid grasp of the genetic foundation of sickle cell disease (SCD). However, there were clear knowledge gaps in the treatment and management of silent cerebral infarcts and what amounts of fluids Clients with SCD should take. The fact that only 18.4% of the nurses were aware of the possibility of stroke from silent cerebral infarcts emphasizes the necessity for focused education in this area, as previously mentioned by Sekome (11). Additionally, only 27.9% of respondents were aware that hydroxyurea is used to raise hemoglobin F levels, a critical treatment for lessening the severity of the condition, even though 81.0% of respondents were aware that routine health exams for Clients with SCD should take place every 6 months. The need for ongoing professional development and training programs to keep nurses up to date on the most recent best practices in managing sickle cell disease is highlighted by this knowledge gap (12). To implement effective preventative measures and customize interventions to the requirements of the population, nurses must possess a solid understanding of the epidemiology of sickle cell disease. The average degree of knowledge highlights the need for improved training and educational endeavors and points to a possible gap.

Nurses’ attitudes towards the management of SCD are influenced by various factors, including concerns about addiction and stigmatization (13). According to the current study findings, nurses’ attitudes had a positive attitude towards the management of pain among clients with SCD. However, some of their perceptions of clients with SCD were not uniform; 36.1% of them thought the clients were looking for drugs. This stigma is not specific to this study; comparable sentiments among healthcare staff have been recorded by Munung et al. (14). Such unfavorable viewpoints can have a detrimental impact on the standard of care given to clients with SCD and increase their hesitancy to seek medical attention. The average attitude score ranged from neutral to negative, highlighting the need for programs that target stigma reduction and enhance nurse-client relationships. This implies that when nurses exhibit empathy, understanding, and positive attitudes towards clients with SCD, it can lead to better client outcomes. Clients who feel more comfortable with nursing care are more likely to adhere to treatment plans and are more open in communicating their symptoms and needs. These findings highlight the importance of addressing negative attitudes and providing additional education and support for healthcare professionals involved in SCD management (14). Furthermore, about 34.0% of respondents expressed that SCD receives less funding and focus for research, which is consistent with the findings of international healthcare literature. Underfunding and neglect of SCD research, according to Lee et al. (15), prevents progress in therapy choices and overall management of the condition. This view may have an impact on nurses’ passion and dedication to SCD treatment, underscoring the necessity of lobbying and legislative measures to improve financing and research for SCD.

According to Lakkakula et al. (16), nurses must initiate and reassess clients’ pain in order to reduce intensity and enhance the quality of care for clients with SCD. According to the study, most nurses followed WHO standards for managing pain and conducted appropriate diagnostic tests. This is encouraging and consistent with the practices documented by Ngonde et al. (8) where for the nurses, fewer than half reported having performed these activities. Nurses are generally competent in the initial identification and diagnosis of SCD, as evidenced by the use of hemoglobin profiling (84.4%) and the diagnosis of at least one SCD case (82.3%). Katawandja et al. (17) discovered a higher practice point range of 40.4 to 87.23%, which is similar to the current study. On the other hand, there were differences in how specific drugs and therapies were used. For instance, only 44.9% of nurses used tramadol and 71.4% used paracetamol to treat SCD pain, compared to 80.3% of nurses who reported using morphine. A high percentage of nurses (84.4%) reported using hydroxyurea, which is known to enhance clinical outcomes in sickle cell disease (SCD) which are reported in another similar study (18). The need for standardized treatment protocols to guarantee consistent and efficient client care is highlighted by this diversity in medication use.

The present study showed a positive relationship between respondents’ age and practice when caring for clients with SCD. It could be that older nurses often have more years of experience and therefore might have more developed skills and knowledge related to SCD management. This experience can translate into better practices and improved client outcomes. Also, understanding that practice improves with age can highlight the importance of retaining older nurses in the workforce and valuing their continued contributions. Furthermore, the findings of this study revealed that knowledge of SCD among respondents have a positive relationship with practice when caring for clients with SCD. This findings is consistent with the findings of Ngonde et al. (8) where for nurses, their SCD knowledge was a significant predictor of their practice in managing the disease. These similarities may be ascribed to the fact that the level of education of the respondents in respective studies are similar. In essence, nurses can improve their practice in managing SCD by increasing their comprehension of the condition and how to manage it through workshops (19, 20). However, the hospital needs to have the tools needed to diagnose SCD in order for this knowledge to be put to practice. In the present study, a negative relationship was observed between respondents’ attitude towards clients with SCD and SCD management practices. These findings contrast that of Jenerette et al. (21) which showed that nurses’ attitudes toward clients with SCD is positively correlated with their practice. This implies that the negative attitudes can result in less compassionate and attentive care. This might manifest as less time spent with clients, inadequate pain management, and a general lack of empathy, all of which can negatively impact client outcomes. Despite the additional advantages of positive practices towards the management of SCD, one must also take into account how negative attitudes towards care for clients with SCD may affect treatment and health care outcomes.

## Limitations of the study

The cross-sectional design used may not allow for generalizability of findings. Other sampling styles may have provided better insights into the knowledge, attitudes and practices of nurses towards SCD management. With that notwithstanding, comparable outcomes using the simple sampling technique were reported by other studies (2, 8). This finding cannot be extended to the full Ghanaian nursing population because other samples from other health care facilities may have provided a far broader picture of the understanding and care practices of nurses towards clients with SCD. Other nurses who were omitted from the study could have increased the sample size for a more understandable picture of the management practices of nurses towards SCD. The study could be prone to various biases such as social desirability and courtesy bias. Also, information on SCD management was self-reported; hence, the chances of reporting bias should not be ignored while interpreting the findings. Data interpretation with knowledge and practice should be considered as other cultural, social, medical and health system factors were not addressed in the current study. The small sample size and cross-sectional nature of the study may prevent generalization of findings and prevent causal relationships, respectively. Future studies could increase the sample size and also include other health care professional perspectives on the management practices towards clients with SCD to better understand and identify predictors of SCD management practices in Ghana. Mixed methods studies could be employed in the future to explain some of the attitudes, misconceptions and practices of nurses towards SCD management to produce more comprehensive findings. Nevertheless, this study is important in understanding the determinants of SCD management practices among nurses as well as the knowledge and attitudes of nurses towards care for clients with SCD.

## Conclusion

Most nurses had low knowledge on SCD and its management. The study also reported a negative attitudes and practice towards the management of clients with SCD. Age, knowledge level and attitudes toward clients with SCD were the factors associated with practice of nurses towards the management of clients with SCD. The study highlights the need for continual education and professional growth of nurses to address knowledge gaps and negative attitudes of nurses towards SCD management, which could affect client care negatively. Hence, nurses should be prepared for the challenges that lie ahead in managing client care issues related to health care, including pain management. Further, new nursing staff should be given hospital orientation programs tailored to individual units, for specialized care and attention to client health care needs. It is imperative for nurses to also pursue specialized nursing education to advance critical knowledge in handling special and emergency conditions requiring prompt nursing and medical interventions. Social, behavior and attitude change communications across the nursing fraternity could aid improve attitude and practices of nurses towards client care and management.

## Data Availability

The original contributions presented in the study are included in the article/Supplementary material, further inquiries can be directed to the corresponding authors.
