# Supplementary material for: Sickle cell disease knowledge, attitudes, and management practices among nurses in northern Ghana: a cross-sectional study
Source: Front Public Health. 2026 Jul 13;14:1811313. doi: 10.3389/fpubh.2026.1811313 (PMC13402410; doi:10.3389/fpubh.2026.1811313)
Supplement: Supplementary file 1 [file Table_1.DOCX]

**APPENDIX I: QUESTIONNAIRE**

**SECTION 1: PARTICIPANT SOCIO-DEMOGRAPHICS**

**1. Gender:**

- Male
- Female

**2. Age:**

- 20-25 years
- 26-30 years
- 31-35 years
- 36-40 years
- 41-45 years
- 46-50 years
- 51-55 years
- 56 years or older

**3. Number of years working as a nurse:**

- Less than 1 year
- 1-5 years
- 6-10 years
- 11-15 years
- 16-20 years
- 21-25 years
- More than 25 years

**4. Highest nursing education attained:**

- Diploma
- Bachelor's degree
- Master's degree
- Doctoral degree

**5. Marital status:**

- Single
- Married
- Divorced
- Widowed

**SECTION 2: KNOWLEDGE ABOUT SICKLE CELL DISEASE**

This section contains 10 multiple choice questions to assess nurses' knowledge about sickle cell disease. Please select the most correct answer for each question.

**1. Sickle cell disease is caused by:**

a) An infection

b) A genetic mutation

c) Poor diet

d) Environmental toxins

**2. The most common symptoms of sickle cell disease include:**

a) Fever, cough, fatigue

b) Joint pain, pallor, jaundice

c) Abdominal pain, nausea, vomiting

d) Anemia, pain crises, infections

**3. How often should patients with sickle cell disease have routine health screenings?**

a) Every 6 months

b) Every 1 year

c) Every 3 years

d) Only when symptomatic

**4. Which medication is most commonly used to manage sickle cell pain crises?**

a) Ibuprofen

b) Morphine

c) Acyclovir

d) Cephalexin

**5. What puts patients with sickle cell disease at increased risk for stroke?**

a) Vaso-occlusive crises

b) Anemia

c) Silent cerebral infarcts

d) Frequent infections

**6. Chronic kidney disease is a common complication of sickle cell disease. True or False?**

**7. What percentage of patients with sickle cell disease have gallstones by age 20?**

a) 10%

b) 30%

c) 50%

d) 70%

**8. How much fluid should patients with sickle cell disease drink daily?**

a) 8 glasses (2 liters)

b) 10 glasses (2.5 liters)

c) 12 glasses (3 liters)

d) No specific recommendation

**9. Hydroxyurea is a medication used to treat sickle cell disease. It works by:**

a) Replacing abnormal hemoglobin S

b) Reducing white blood cell counts

c) Increasing hemoglobin F levels

d) Improving red blood cell hydration

**10. All of the following can trigger a sickle cell pain crisis EXCEPT:**

a) Dehydration

b) Stress

c) Cold temperatures

d) Exercise

**SECTION 3: ATTITUDES TOWARDS SICKLE CELL DISEASE**

This section contains statements about the attitudes towards patients with sickle cell disease. Please indicate your level of agreement with each statement

1. Patients with sickle cell disease are sometimes perceived as drug-seeking.

o Strongly agree

o Agree

o Not sure

o Disagree

o Strongly Disagree

2. Sickle cell disease is often associated with low socioeconomic status.

o Strongly agree

o Agree

o Not sure

o Disagree

o Strongly Disagree

3. Patients with sickle cell disease have a tendency to be noncompliant with treatment.

o Strongly agree

o Agree

o Not sure

o Disagree

o Strongly Disagree

4. Sickle cell disease primarily affects African Americans.

o Strongly agree

o Agree

o Not sure

o Disagree

o Strongly Disagree

5. There are negative stereotypes associated with sickle cell disease.

o Strongly agree

o Agree

o Not sure

o Disagree

o Strongly Disagree

6. Sickle cell disease receives less research funding and attention compared to other genetic

diseases.

o Strongly agree

o Agree

o Not sure

o Disagree

o Strongly Disagree

7. Discrimination against patients with sickle cell disease still exists in the healthcare system.

o Strongly agree

o Agree

o Not sure

o Disagree

o Strongly Disagree

8. I make a conscious effort to avoid stereotyping patients with sickle cell disease.

o Strongly agree

o Agree

o Not sure

o Disagree

o Strongly Disagree

**SECTION 4 :Nurses’ Practice Related to Diagnosis, Crisis Management and Outpatient Follow-Up of Sickle Cell Patients**

| Statement | Yes | No |
| --- | --- | --- |
| **Screening or diagnosis** |  |  |
| Haemoglobin profile |  |  |
| Practitioner diagnosed at least one case of SCD |  |  |
| **Symptoms or signs evoking diagnosis** |  |  |
| Fever |  |  |
| Pain |  |  |
| Cutaneous-mucosal pallor |  |  |
| Notion of previous transfusion |  |  |
| Jaundice |  |  |
| Swelling of hands and feet in infants |  |  |
| Notion of multiple deaths in the immediate family |  |  |
| **Assessment of pain** |  |  |
| Subjective (based on patient complaint) |  |  |
| Using a scale |  |  |
| **Use of usual analgesics** |  |  |
| Empirically |  |  |
| According to WHO guidelines |  |  |
| **Analgesics used** |  |  |
| Paracetamol |  |  |
| Paracetamol and codeine |  |  |
| NSAIDs (diclofenac, ibuprofen…) |  |  |
| Tramadol |  |  |
| Morphine |  |  |
| **Other treatments** |  |  |
| Transfusion |  |  |
| Hydration with saline |  |  |
| Oxygen |  |  |
| Hydroxyurea |  |  |
| **Outpatient follow-up** |  |  |
| Provider has ever followed a SCD patient |  |  |
| SCD follow-up may be once a month |  |  |
| **Laboratory tests** |  |  |
| Haemoglobin. white blood cell count. complete blood count |  |  |
| Sedimentation rate |  |  |
| C-reactive protein |  |  |
| Lactate dehydrogenase |  |  |
| Urea and creatinine |  |  |
| Transaminases |  |  |
| Total/indirect bilirubin |  |  |
| 10-reagent urine dipstick |  |  |
| Conventional radiography |  |  |
| Abdominal ultrasound |  |  |
| Cardiac ultrasound |  |  |
| Transcranial doppler ultrasound |  |  |
| Electrocardiogram |  |  |
| CT scan |  |  |
| **Reason for transfer** |  |  |
| Medical logistic and financial |  |  |
| Limited clinical skills of the centre |  |  |
| **Medical reasons for referral** |  |  |
| Pain resistant to usual analgesics |  |  |
| Persistent infection |  |  |
| Decompensated anaemia |  |  |
| **Reference-counter-reference** |  |  |
| Provider develops referral score |  |  |
| Provider receive counter-reference score |  |  |

**SCD, sickle cell disease; WHO, World Health Organizations; CT, computed tomography; NSAIDs, Non-steroidal anti-inflammatory drugs.**
